# Congenital Cardiovascular Disease for the General Radiologist: More Common Than you Think!

**DOI:** 10.5334/jbr-btr.1037

**Published:** 2016-02-09

**Authors:** Daniel GH. Devos

**Affiliations:** 1 UZ Gent, BE

Every thoracic computer tomography (CT) image dataset encompasses the heart. Nevertheless, it is often disregarded in theradiologist’s report; especially congenital heart disease may go unreported. We present a number of congential cardiac conditions that can be detected even on untriggered CT images.

Abnormal coronary origin and course (between pulmonary trunc and aortic root), although not very common, is responsible for sudden cardiac death as a presenting symptom in a retrospective postmortem series of congenital heart disease; mostly young people, and many of them athletes, are affected.

Intramyocardial course and myocardial bridging of mainly left anterior descending coronary arteryare more difficult to connect to cardiac symptoms or even to cardiac sudden death. It is, however, quite easy to detect even on transverse images by the typical ‘coronary dive’. It can explain worrying electrocardiographic-abnormalities, as well as angina.

A bicuspid aorticvalve (BAV) is very difficult to pick up on transverse CT images, but easy to depict in the proper reconstruction planes, especially in comparison with the occasionally very challenging imaging by ultrasound. It is emphasized that BAV is rarely a lesion on its own: beware for ascending aortic dilation, aortic coarctation, and even left ventricular cardiomyopathy.

A persistent left superior vena cava should be noted and reported. It can seriously ruin power bolus injection for CT imaging. It can equally complicate positioning of a Schwan-Ganz catheter or pacemaker/Implanted Cardioverter Device (ICD) leads, due to absence of the innominate vein. Rarely a left-to-right shunt or more complex congenital heart disease is present.

Thoracic CT can help solve the conundrum of an unexplained dilated right ventricle, where obvious causes suc h as pulmonary emboli and arrhythmogenic right ventricular cardiomyopathyhave been ruled out. An abnormal pulmonary venous return or a sinus venosus defect, for example, can easily be found.

A dilated aortic root in a young patient or an aortic dissection in a young, tall and slender patient should raise suspicion of MarfanSyndrome, which is important because the therapeutic approach of aortic dissection in Marfan syndrome is always surgical. Allthough Marfan syndrome is classified as a connective tissue disease, it should be borne in mind that it affects the heart and aorta of patients from an early age on.

In contrast to CT, Magnetic Resonance is a powerfull tool to quantify volumes and shunts in known congenital heart disease. With the aformentioned lesions in mind, the radiologist will be capable of reporting congential cardiovascular lesions that may have importance for the patient, either for the query at hand, orto anticipate symptoms or problems later in life.

## Competing Interests

The author declares that they have no competing interests.

